# Role of quetiapine beyond its clinical efficacy in bipolar disorder: From neuroprotection to the treatment of psychiatric disorders (Review)

**DOI:** 10.3892/etm.2015.2213

**Published:** 2015-01-23

**Authors:** MÁRCIO G. SOEIRO-DE-SOUZA, VASCO VIDEIRA DIAS, GIOVANNI MISSIO, VICENT BALANZÁ-MARTINEZ, LEANDRO VALIENGO, ANDRÉ F. CARVALHO, RICARDO ALBERTO MORENO

**Affiliations:** 1 Mood Disorders Unit (GRUDA), Institute of Psychiatry, University of São Paulo, São Paulo, Brazil; 2 University Hospital Doctor Peset and Section of Psychiatry, University of Valencia, Valencia, Spain; 3 CIBER Mental Health (CIBERSAM), Carlos III Health Institute, Madrid, Spain; 4 Laboratory of Neuroscience (LIM27), Institute of Psychiatry, University of São Paulo, São Paulo, Brazil; 5 Psychiatry Research Group and Department of Clinical Medicine, Federal University of Ceará, Fortaleza, Brazil

**Keywords:** quetiapine, depression, neurocognition, antipsychotics, neuroprotection, psychopharmacology

## Abstract

The aim of the present review was to discuss the following aspects of treatment with quetiapine in psychiatric disorders: i) Neurocognition and functional recovery in bipolar disorder (BD); ii) neuroprotective profile in different models; and iii) potential off-label indications. A PubMed search was conducted of articles published in English between 2000 and 2012 on quetiapine, cross-referenced with the terms ‘anxiety’, ‘attention deficit disorder’, ‘borderline personality disorder’, ‘dementia’, ‘insomnia’, ‘major depressive disorder’ (MDD), ‘obsessive-compulsive disorder’, ‘post-traumatic stress disorder’, ‘remission’, ‘cognition’, ‘neurobiology’, ‘neuroprotection’, ‘efficacy’ and ‘effectiveness’. Articles were selected from meta-analyses, randomized clinical trials and open trials, and the results were summarized. Quetiapine, when studied in off-label conditions, has shown efficacy as a monotherapy in MDD and general anxiety disorder. Quetiapine also appears to exhibit a small beneficial effect in dementia. The review of other conditions was affected by methodological limitations that precluded any definitive conclusions on the efficacy or safety of quetiapine. Overall, the present review shows evidence supporting a potential role for quetiapine in improving cognition, functional recovery and negative symptoms in a cost-effective manner in BD. These benefits of quetiapine are potentially associated with its well-described neuroprotective effects; however, further studies are clearly warranted.

## 1. Introduction

Bipolar disorder (BD) is one of the leading causes of disability in the world (1,2). Following initial optimistic estimations about the outcome of the disorder, it has become clear that substantial morbidity, functional deficits and poor quality of life persist in the majority of individuals with BD (3–5).

Quetiapine promotes an increase in prefrontal dopamine release through the antagonism of 5-hydroxytryptamine (5-HT)_2A_ receptors, partial agonism of 5-HT_1A_ receptors and antagonism of α2 adrenoceptors (6). Quetiapine additionally augments serotoninergic transmission by increasing the density of 5-HT_1A_ receptors in the prefrontal cortex and though the antagonism of 5-HT_2A_ receptors and α2 adrenoceptors. By comparison, norquetiapine, the primary active metabolite of quetiapine, functions as a 5-HT_2C_ receptor antagonist and a potent inhibitor of the norepinephrine transporter (NET). As a result of NET inhibition, the concentration of norepinephrine in the synapse increases, which, together with the increase in prefrontal dopamine and serotonin levels, could explain the efficacy of quetiapine as an antidepressant across several clinical trials (3,7). Norquetiapine also blocks presynaptic 5-HT_7_ receptors, which may contribute to the overall antidepressant effects (8,9), and quetiapine and norquetiapine act as antagonists at dopamine D2 receptors with moderate affinity (10,11). These properties, and evidence of efficacy against positive, negative, cognitive and affective symptoms, have led to the recognition of quetiapine as a well-established, atypical antipsychotic treatment for patients with schizophrenia (12–14). In BD, quetiapine is the only antipsychotic medication with evidence of efficacy across all phases (15,16). Such evidence includes large-scale trials demonstrating efficacy in acute bipolar mania (17–19), as well as in maintenance treatment (20–22). Furthermore, studies have shown that quetiapine is effective in reducing depressive symptoms both in monotherapy and in adjunctive therapy for patients with major depressive disorder (MDD) who have shown an inadequate response to antidepressant treatment (23–27). In this regard, quetiapine is now approved by the US Food and Drug Administration (FDA) as an adjunctive treatment for patients with MDD following inadequate response to standard antidepressants. In BD depression, quetiapine has been demonstrated to improve anxiety symptoms, quality of life and quality of sleep (28–30). Furthermore, quetiapine has been reported to improve quality of life in all forms of mood disorders (31,32).

Quetiapine is a pleiotropic agent that affects multiple neurotransmitter and non-transmitter targets (9). This drug may therefore exhibit efficacy for the management of a wide range of mental disorders. Accumulating evidence suggests that quetiapine may be efficacious for the management of several disparate mental disorders, including MDD (33,35–39), generalized anxiety disorder (GAD) (34,40–44), borderline personality disorder (BPD) (34,45,46) and obsessive-compulsive disorder (OCD) (47,48).

The present review discusses the evidence for quetiapine as a treatment for disparate mental disorders. Furthermore, the potential neuroprotector and/or neurotrophic effects of quetiapine, which may contribute to its efficacy across a wide range of mental disorders along with possible pro-cognitive effects, are explored. Since it has previously been established that quetiapine is an efficacious agent for BD and schizophrenia (12,49), these data are not covered in detail in the present review.

## 2. Literature review

A PubMed search was conducted of articles published in English between January 1, 2000 and December 31, 2012 on quetiapine, cross-referenced with the terms ‘anxiety’, ‘attention deficit disorder’, ‘borderline personality disorder’, ‘dementia’, ‘depression’, ‘insomnia’, ‘major depressive disorder’, ‘obsessive-compulsive disorder’ and ‘post-traumatic stress disorder’. Furthermore, the terms ‘response’, ‘remission’, ‘cognition’, ‘neurobiology’, ‘neuroprotection’, ‘efficacy’ and ‘effectiveness’ were matched with the term ‘quetiapine’. Articles were selected from meta-analyses, randomized clinical trials (RCTs) and open trials, and the results were summarized. The aim of the present study was to review the following aspects of treatment with quetiapine in psychiatric disorders: i) Neurocognition and functional recovery in BD; ii) neuroprotective profile in different models; and iii) potential indications.

## 3. Profile of quetiapine with regard to neurocognition and functional recovery in subjects with BD

Although first-generation antipsychotics (FGAs) have previously been demonstrated to exert few or no beneficial effects on cognitive impairment, this view has subsequently been challenged (50,51). Second-generation antipsychotics (SGAs), by contrast, have been associated with a wide range of therapeutic effects beyond just antipsychotic efficacy, and their benefits on cognition are apparent (52,53). A caveat, however, is that unmedicated patients have better verbal fluency and memory performances than patients medicated with atypical antipsychotics (54–56).

Quetiapine has demonstrated superior neurocognition-enhancing properties in several RCTs of schizophrenia when compared with olanzapine (57–60) or risperidone (57–59), a finding supported by a meta-analysis (61). By contrast, other studies have demonstrated a comparable neurocognitive efficacy of quetiapine, olanzapine and risperidone (45,46,62–64). In studies of schizophrenia, the cognitive benefits of quetiapine have been attributed to its loose binding to and fast dissociation from dopamine D2 receptors (60,65). In these studies, patients with schizophrenia and/or schizoaffective disorder were randomized to treatment with quetiapine (typical mean doses of ~600 mg/day) or other SGAs (37–39). A study by Harvey *et al* in 2006 (64) randomized patients with schizophrenia, diagnosed according to the Diagnostic and Statistical Manual of Mental Disorders (fourth edition) criteria, to receive either risperidone (mean dose, 5.33 mg/day; n=154) or quetiapine (mean dose, 529.6 mg/day; n=135) for eight weeks. By the end of the trial both drugs enhanced performance-based social competence, and no significant differences were found between the groups (64). In a study by Robles *et al* (64), patients with first-episode psychosis were randomized to quetiapine (mean dose, 532.8 mg/day; n=24) or olanzapine (mean dose, 9.7 mg/day; n=26) treatment groups for six months. A neurocognitive battery was administered at baseline and at the end of the trial. No improvement in cognition was observed following SGA treatment and no statistically significant differences were found between groups at the endpoint of the study (64). Overall, these RCTs indicate that quetiapine improves cognitive functioning in patients with schizophrenia; however, methodological heterogeneity (e.g. in recruited samples) across studies does not allow comparisons between quetiapine and other SGAs regarding cognitive effects.

Although it has been suggested that SGAs may improve cognitive functioning in schizophrenia, this may not be the case in BD, where antipsychotics have shown more negative effects on cognition than lithium and anticonvulsants (66,67). In an RCT with a cross-over design, the acute effects of risperidone (2 mg) or quetiapine (200 mg) were assessed in patients with stable BD type I. Quetiapine was associated with more immediate adverse overall cognitive performance and sedation than risperidone (68). Conversely, Torrent *et al* (55) reported that, compared with olanzapine (mean dose, 7.7 mg/day) and risperidone (mean dose, 3.7 mg/day), euthymic patients with BD treated with quetiapine (mean dose, 404.1 mg/day) showed a better performance in learning, short-term memory and recognition tasks assessed with the California Verbal Learning Test, as well as in verbal fluency (55); however, this study was naturalistic, and euthymic patients with BD treated with SGAs have been shown to perform worse than stable patients treated with standard mood stabilizers (37). In summary, treatment with SGAs may be associated with adverse cognitive effects in BD, partly due to their sedative properties.

Functional recovery is defined in terms of several different behavioral domains, including social, occupational, educational and independent living. Quetiapine treatment has been associated with symptomatic remission, syndromal recovery and improvements in quality of life (69,70); however, the magnitude of these beneficial effects of quetiapine is an area of active research. The majority of the earlier prospective follow-up studies of BD focused on relapse and residual symptoms rather than on functional outcome (71). In addition, these prospective studies highlighted the fact that syndromal remission often lagged behind functional recovery. Functional recovery is not only about an absence of symptoms, but also the recovery of independence regarding daily activities and professional and social life. Further studies with quetiapine and other atypical antipsychotics in this area are warranted.

BD also has a significant impact upon a patient’s quality of life, imposing a considerable economic burden on the individual, family members and society as a whole. Although several medications are indicated for the acute treatment of mania and depression associated with BD, as well as for maintenance therapy, these drugs have varying efficacy, tolerability and costs (72,73).

Despite the fact that the efficacy of antipsychotics as a maintenance treatment in BD has not been systematically studied, their use is frequently observed in the long-term treatment of BD, and it is not unusual for a patient with BD to adhere to a regimen comprising three to four medications, including antipsychotics. Conventional antipsychotics may have comparable efficacy to lithium for acute mania, but limitations arise when they are used in the long-term treatment of BD. A number of adverse effects have been associated with conventional antipsychotics, including extrapyramidal symptoms (EPSs), tardive dyskinesia, weight gain, sedation and sexual dysfunction, and these often lead to non-compliance. The use of conventional antipsychotics may have a negative effect on the overall course of the illness, and these drugs may exhibit an inferior efficacy to lithium in the treatment of the core manic symptoms over a long period of time (74–77). By contrast, atypical antipsychotics have a favorable side effect profile, good thymoleptic properties and positive effect on overall functioning, and may therefore prove useful for patients with BD who require antipsychotic treatment (74,75).

The successful treatment of patients with mood disorders exhibiting psychotic symptoms is a complex challenge. The administration of antipsychotic medications in these individuals may be associated with EPSs, enhanced depression and functional impairment. Treatment with typical antipsychotics, such as quetiapine, is associated with a decreased incidence of adverse events (AEs), such as EPSs (77–79).

According to a systematic review and meta-analysis (80), most monotherapy drug regimens (MDRs), when compared with a placebo, showed significant response and remission rates in acute mania. In cases of bipolar depression, however, only quetiapine and, to a lesser extent, olanzapine demonstrated efficacy as an MDR. Overall, patients tolerated MDRs well, and there were low discontinuation rates due to any cause or AEs, although the AE profiles differed among the treatments. The majority of the MDRs were efficacious and safe in the treatment of manic episodes, but few MDRs demonstrated equivalent efficacy to quetiapine in the treatment of depressive episodes in BD (80,81).

A recent cost effectiveness analysis of quetiapine conducted by Ekman *et al* in 2012 (73) found that, for a patient starting with acute depression or in remission at 40 years of age (the average age in the clinical trials), the use of quetiapine at 300 mg/day was a more cost-effective strategy than that of olanzapine at 15 mg/day over a five-year time frame (73). The authors concluded that, compared with olanzapine, quetiapine is cost effective as a maintenance treatment for BD.

## 4. Quetiapine and its potential neuroprotective effects in BD and implications for other psychiatric disorders

Regarding preclinical studies *in vitro*, quetiapine has shown neuroprotective effects through a wide range of biological targets (82). In the antioxidant system, quetiapine has been demonstrated to protect against the inhibition of the antioxidant enzyme superoxide dismutase (SOD) and against hydrogen peroxide-induced pro-oxidant effects *in vitro* (42,82). Quetiapine has additionally been reported to protect cultured cells against oxidative stress-related cytotoxicity induced by amyloid-β (Aβ) (42,43), as well as decrease the size of Aβ plaques in the brains of amyloid precursor protein/presenilin-1 double-mutant mice. These findings support an important role of quetiapine in decreasing intracellular reactive oxygen species (ROS) and calcium levels, thus contributing to its antioxidant properties (83,84). Quetiapine has also been shown to prevent the Aβ-induced overproduction of intracellular ROS (84) and decreases in the levels of the antioxidant enzymes SOD, catalase and glutathione peroxidase (84,85).

In the immune system, quetiapine significantly blocks tumor necrosis factor-α release from activated microglia and nitric oxide production, which may indirectly protect neurons and facilitate neurogenesis and synaptic activity (86,87). Furthermore, quetiapine has been reported to have anti-inflammatory effects in a murine collagen-induced arthritis model (88,89).

Other findings on the neuroprotective effects of quetiapine include its ability to reverse the suppression of chronic stress-induced hippocampal neurogenesis (90–92), an effect that appears to be synergistic when using antidepressants (2). Similarly, quetiapine improves vascular depression symptoms in both human and preclinical models (3–5). Recently, Bi *et al* (3) showed that two weeks of pretreatment with quetiapine in an animal model of vascular depression limited myelin breakdown and oligodendrocyte loss, and subsequently increased oligodendrocyte maturation (3). These results support a potential role for quetiapine in the prevention of oligodendrocyte damage through white matter neuroprotection in vascular depression.

In terms of neurotrophic factors, quetiapine has been shown to enhance hippocampal brain-derived neurotrophic factor (BDNF) and fibroblast growth factor-2 expression (8,9). In addition, chronic treatment with quetiapine attenuates the decrease in cortical and hippocampal BDNF expression that occurs in different animal models of depression (10,11). In other preclinical studies, quetiapine has been shown to increase the number of mature oligodendrocytes in demyelinated lesions by decreasing expression of the transcription factor olig2 and improve spatial working memory (12–14). Similar findings, namely the reduction of cuprizone (a copper chelator)-induced white matter demyelination by quetiapine, have also been described (15,16).

In human studies, quetiapine extended-release treatment (300 mg/day) for 16 weeks has been shown to increase BDNF levels in depression and to decrease levels in mania/mixed episodes (17–19). Similar findings were also observed in other clinical studies (20). In depressive episodes in MDD and BD, increased plasma BDNF levels were observed following the addition of a low dose of quetiapine and other atypical antipsychotics to standard antidepressants for four weeks (20–22). Quetiapine treatment has similarly been associated with a significant increase in neuropeptide Y and a decrease in corticotropin-releasing factor levels, and these changes predicted clinical response in patients with schizophrenia (23–27). Padurariu *et al* (28) observed an increase in SOD activity in subjects with schizophrenia treated with quetiapine and haloperidol (28). Finally, in imaging studies, anterior cingulate cortex myo-inositol levels have been shown to be associated with antidepressant response to quetiapine in adolescents with bipolar depression (31,32). The present findings therefore suggest an important role for quetiapine in the activation of neuroprotective pathways in diverse models, thus providing support to its therapeutic role in several psychiatric disorders.

## 5. Potential off-label indications of quetiapine in other psychiatric disorders

Although not approved by regulatory agencies such as the FDA, quetiapine has been used for the treatment of psychiatric conditions other than BD. The most frequent off-label uses of quetiapine by physicians are the treatment of attention deficit hyperactivity disorder (ADHD), BPD, dementia, insomnia, MDD, OCD, post-traumatic stress disorder (PTSD) and other anxiety disorders,

### ADHD and conduct disorder

A number of studies have shown a possible role of quetiapine in conduct disorder (33–34). In a seven-week, placebo-controlled RCT with 19 youths, the experimental group, which received 200–600 mg/day quetiapine, showed significant improvements in all clinical scales compared with the placebo group (33). An open-label study also demonstrated the potential benefit of add-on quetiapine for ADHD and conduct disorder (34). Quetiapine has been shown to be effective in reducing ADHD symptoms and aggression in individuals who did not respond sufficiently to an MDR with methylphenidate at a dose of 54 mg/day (34,45,46). Although these studies have shown positive results regarding conduct disorder and ADHD with few adverse effects, the evidence for the use of quetiapine in these conditions is clearly limited due to the small number of controlled studies in this area.

### PTSD

PTSD is a complex disorder generally co-occurring with other conditions, such as psychosis and substance use, mood and anxiety disorders. A number of studies have evaluated the potential role of quetiapine in the treatment of PTSD (47,48). One open-label study (50) with 53 patients found that eight weeks of monotherapy with quetiapine reduced the majority of the psychotic and PTSD symptoms classified using the Clinician-Administered PTSD Scale (CAPS) and the Positive and Negative Syndrome Scale, and increased the Clinical Global Impression-Improvement Scale (CGI-I) scores. Another open study (52), showed that a modest dose of quetiapine as an add-on to a serotonin-specific reuptake inhibitor (SSRI) provided significant relief from PTSD symptoms with a 42% overall improvement based on the CAPS and significant improvements in each dimension of symptoms: Re-experiencing, hyperarousal and avoidance. Other open studies showed similar results (54–56).

The design of RCTs with larger sample sizes is required to better define the potential role of quetiapine in the treatment of PTSD, but open-label studies have suggested that quetiapine may be efficacious for the management of treatment-resistant PTSD.

### BPD

BPD is characterized by intense, rapidly fluctuating mood states combined with impulsivity and interpersonal difficulties (57–60). The standard treatment consists of certain forms of specific psychotherapy, but most of these require treatment for a year or longer, which may be costly (57–59). Atypical antipsychotics have been used off-label for treating symptoms associated with BPD, such as impulsivity and agitation. Furthermore, BPD frequently coexists with depressive and anxiety disorders. Based on its mechanisms of action, quetiapine may help to treat these disorders in patients in whom first-line treatment is unsuccessful. A number of open studies have investigated the role of quetiapine in BPD. The control of impulsivity in BPD by quetiapine was assessed and showed promising results with few adverse effects; the adverse effects observed were predominantly sedation and weight gain (45). These studies showed beneficial results on several symptom scales.

Several studies have additionally suggested the potential benefit of quetiapine in other features of BPD, such as affective symptoms (45,46,62–64), and have suggested it may result in an improvement in cognitive function (60,65). It has been shown that the executive and verbal cognitive test scores of patients with BDP decreased following treatment with quetiapine (66,67). In summary, despite there being a number of open studies suggesting certain benefits of quetiapine in BPD, RCTs with larger sample sizes are necessary to assess the true potential of this agent in this group of patients.

### Dementia

Behavioral symptoms may appear in mild dementia and become more severe with the progression of the disease (93). Agitation, aggressiveness and psychosis can be observed in Alzheimer’s disease and in other types of dementia (93). These behavioral disturbances are more often the cause of the institutionalization of these patients due to the heavy assistance and emotional burden they represent for caregivers (93). Traditionally, these symptoms were controlled by FGAs, which, following long-term use, have been associated with severe EPSs, sedation, orthostatic hypotension and cognitive dysfunction. More recently, atypical antipsychotic agents have begun to be used based on a better tolerability profile and a reduced incidence of the aforementioned side effects.

In a 36-week, multi-centric, placebo-controlled RCT (90)with 421 outpatients with Alzheimer’s disease and psychosis, aggression or agitation, the patients were randomly assigned to receive olanzapine (mean dose, 5.5 mg/day), quetiapine (mean dose, 56.5 mg/day), risperidone (mean dose, 1.0 mg/day) or a placebo. No significant differences were found among the treatments with regard to the time to their discontinuation for any reason (medians of 8.1, 5.3, 7.4 and 8.0 weeks for olanzapine, quetiapine, risperidone and the placebo, respectively; P=0.52). The median time to treatment discontinuation due to inefficacy favored olanzapine (22.1 weeks) and risperidone (26.7 weeks) as compared with quetiapine (9.1 weeks) and placebo (9.0 weeks) (P=0.002), whereas the time to treatment discontinuation due to AEs or intolerability favored the placebo. Overall, 24% of patients who received olanzapine, 16% of patients who received quetiapine, 18% of patients who received risperidone and 5% of patients who received the placebo discontinued their assigned treatment due to intolerability (P=0.009). With regard to improvement on the CGI scale, no significant differences were observed among the groups. The adverse effects observed in the study limited the advantage of the efficacy of atypical antipsychotic drugs for the treatment of psychosis, aggression or agitation in patients with Alzheimer’s disease. Furthermore, atypical antipsychotics were associated with a decline in cognitive function at a magnitude consistent with one year of deterioration compared with the placebo. Further cognitive impairment should therefore be considered as an additional risk of treatment with atypical antipsychotics when treating patients with Alzheimer’s disease (71).

There are several RCTs regarding the use of quetiapine to treat agitation and psychosis associated with dementia, with contradictory results (74–77). The studies with negative results are those with small sample sizes (74,75); however, the majority of the studies are consistent in the high rate of adverse effects, such as sedation and orthostatic hypotension. Despite this, when comparing the use of quetiapine, haloperidol and a placebo control, both active treatments had positive results on agitation, with fewer adverse effects observed with quetiapine (77–79). In the inpatient clinic, the effects of rivastigmine, quetiapine and a placebo on the alleviation of agitation were compared; all treatments were equal to quetiapine regarding cognitive decline (94).

In a meta-analysis comparing the effects of quetiapine with those of a placebo (81), improvements in the neuropsychiatry inventory (NPI) and CGI-I scores were observed in the patients receiving quetiapine when compared with those receiving the placebo, with a weighted mean difference of −3.05 [95% confidence intervals (CI), −6.10–−0.01) and −0.31 (95% CI, −0.54–−0.08), respectively. The meta-analysis found that quetiapine exhibited a statistically higher efficacy than the placebo in the treatment of the behavioral symptoms associated with dementia, as measured by the NPI and CGI-I scales. Despite this, the improvement was of a small magnitude and the observable clinical significance is questionable.

In summary, the effect of quetiapine in controlling the behavioral symptoms of dementia is most likely small. Furthermore, the drug should be used with caution due to the possible appearance of severe adverse effects. There is additionally a requirement for clinicians to consider the fact that antipsychotics have been associated with an increased risk of mortality when used in elderly patients treated for dementia-related psychosis, according to the FDA warning.

### Other anxiety disorders

Several studies have examined quetiapine in anxiety disorders, particularly GAD. In this group of patients, quetiapine is generally used either in monotherapy for uncomplicated GAD or as augmentation agent for SSRIs in refractory GAD. The use of quetiapine as a monotherapy for GAD is well documented with several placebo-controlled RCTs (41–45). These studies all revealed positive results on anxiety using the Hamilton Anxiety Rating Scale (HAM-A) with short-term administration periods (four to seven days) and extending up to nine weeks. The dose of quetiapine ranged from 50 to 300 mg/day.

One study in which quetiapine extended-release (XR) was administered in different doses (50, 150 and 300 mg/day) and compared with a placebo treatment showed discontinuation rates of 13.2, 16.5, 24.0 and 5.4%, respectively, due to an AE; 1.9, 1.4, 3.7 and 1.8% of patients experienced clinically significant changes in glucose levels (42).The most common AEs associated with quetiapine XR included dry mouth, somnolence, sedation and constipation (42,43). With regard to the use of quetiapine as an augmentation therapy for SSRIs in refractory patients, there are fewer studies and overall their results are negative (83,84).

One meta-analysis of four studies, including 1,383 patients with GAD, indicated that treatment with 150 mg quetiapine was more likely to lead to a clinical response (relative risk, 1.31; 95% CI, 1.20–1.44), remission and a greater decrease in the HAM-A score than treatment with the placebo (83). There was, however, an increased risk of all-cause discontinuation and weight gain with quetiapine (83).

Regarding other anxiety disorders, an eight-week study showed positive results for social anxiety disorder with quetiapine (86,87). The Brief Social Phobia Scale (BSPS) and the CGI-I were the primary outcome measures, while the Social Phobia Inventory (SPIN) and the Sheehan Disability Inventory (SDI) were secondary outcome measures. No significant difference in the BSPS score at the study endpoint was observed between the quetiapine and placebo groups. Significant time effects were reported for the SPIN and SDI, as well as a significant time versus treatment effect in favor of quetiapine on the SPIN. Quetiapine was additionally shown to have a larger effect on the SPIN. In summary, quetiapine monotherapy appears to be more efficacious than placebo treatment for uncomplicated GAD; however, issues with adverse effects and tolerability may limit its use.

### Insomnia

The sedative effect associated with quetiapine has favored its prescription for insomnia. The single placebo-controlled RCT for primary insomnia with 13 patients (quetiapine dose, 25 mg/day) (88) did not show a difference between the treatment and control groups. In two other studies, one RCT and an open trial, low doses of quetiapine were associated with improved sleep quality in patients with fibromyalgia (89,91).

### OCD

A total of 7 studies using quetiapine for the treatment of OCD were performed, all of them as an add-on therapy (48,96–101). Six of the studies were double-blind RCTs and eight were open trials. The majority of the studies recruited patients with refractory OCD and all used quetiapine as an augmentation therapy for antidepressants. Although the results were contradictory, the studies with refractory patients usually had negative results and vice-versa. A number of trials compared quetiapine with another antipsychotic. For instance, Diniz *et al* (96) reported significant decreases in the Yale-Brown Obsessive Compulsive Scale (Y-BOCS) scores with quetiapine as an add-on therapy but not with clomipramine augmentation. Another study (48) showed improvement in 80% of the quetiapine group and in 44.4% of the ziprasidone group, with an overall mean improvement on the Y-BOCS scale of 66.7%. The mean Y-BOCS and CGI scores were higher in the ziprasidone group at the two-, three- and six-month follow-up points than those in the quetiapine group. One open study with postpartum females showed quetiapine to be effective for OCD (102). The main issue in almost all studies was the small sample size, thus limiting their findings.

Two meta-analyses investigated the use of quetiapine and other atypical antipsychotics in a range of psychiatry disorders (16,102). It was reported that quetiapine monotherapy was not typically effective for OCD, but that its combination with another standard treatment had the potential to provide a therapeutic benefit.

### MDD

MDD or unipolar depression is a common condition with a lifetime prevalence of ≤20%, and leads to considerable suffering and disability (103). Certain antipsychotics have been reported to induce remission in MDD when added to an antidepressant. Similar to anxiety disorders, the use of quetiapine in MDD can be via administration as a monotherapy or as an add-on to antidepressants. The latter is already an indication for the use of quetiapine according to the FDA, but not yet for the European Medicines Evaluation Agency.

Several RCTs and a number of meta-analyses of the use of quetiapine in MDD have been carried out (35–39). In patients with MDD, quetiapine XR (150 and 300 mg/day) monotherapy reduced depressive symptoms, with significant improvements compared with the placebo from between days 4 and 7 (25,36) and up to eight weeks (40). The efficacy of maintenance treatment with quetiapine XR (50–300 mg/day) was demonstrated in a 52-week, placebo-controlled, double-blind RCT (39) consisting of 776 patients stabilized on quetiapine XR (Montgomery-Åsberg Depression Rating Scale score ≤12 and CGI-severity score ≤3), who were further randomized to receive quetiapine XR or a placebo. The first group exhibited a longer time for the recurrence of a depressive event from randomization.

Another RCT (104) consisting of three arms (duloxetine, quetiapine XR and placebo) for MDD showed that the two active treatments were effective for maintenance treatment; the response to quetiapine was earlier than that to duloxetine, occurring in weeks one and two, respectively. Treatment-associated AEs were dry mouth, sedation and somnolence for quetiapine XR and nausea, headache, dizziness and dry mouth for duloxetine (104). In adjunctive therapy with antidepressants, quetiapine was found to be effective between weeks one and eight (38,105–107). In another study, the combination of quetiapine with cognitive-behavioral therapy (CBT) was found to be superior to placebo plus CBT (108).

A different meta-analysis evaluating the efficacy of atypical antipsychotics in MDD showed positive results (38). Compared with the placebo, quetiapine monotherapy [three RCTs; response, n=1,342; odds ratio (OR), 0.52; 95% CI, 0.41–0.66] and quetiapine augmentation (two RCTs; response, n=937; OR, 0.68; 95% CI, 0.52–0.90) showed symptom reduction. In this meta-analysis, aripiprazole, quetiapine and, to a lesser degree, olanzapine and risperidone augmentation were found to have beneficial effects compared with the placebo (38).

## 6. Discussion and conclusion

This review has demonstrated that quetiapine has therapeutic potential for the treatment of several mental disorders. Its pleiotropic mechanism of action may at least partly explain its broad efficacy. There are, however, limitations in the available evidence for several indications.

Quetiapine may be effective for the treatment of core features of BPD, including affective instability, impulsivity and agitation/aggression; however, the evidence originates from uncontrolled observations. There are well-designed RCTs for other atypical antipsychotics, including ziprasidone (109), olanzapine (110,111) and aripiprazole (112). Future RCTs are required to confirm the efficacy of quetiapine for the management of BPD.

Several studies have tested the efficacy of quetiapine for the management of anxiety disorders. Quetiapine may be efficacious for the treatment of refractory PTSD; however, the evidence is derived from open-label trials. Data from add-on RCTs of quetiapine augmentation for treatment-resistant OCD are contradictory. The most compelling evidence is for quetiapine monotherapy for the treatment of GAD. Several RCTs (41–44) and a meta-analysis (83) support its overall efficacy in monotherapy for GAD. Notwithstanding its efficacy, several safer agents are available for the treatment of GAD, including selective serotonin and noradrenaline reuptake inhibitors, e.g. venlafaxine (113). Even low doses of quetiapine, such as 150 mg/day, which are commonly used for the management of anxiety disorders, may lead to weight gain, sedation and metabolic consequences (19). These aspects should be balanced prior to considering quetiapine monotherapy for the management of anxiety disorders.

Quetiapine appears to be efficacious for the treatment of agitation in patients with Alzheimer’s disease and dementia; however, the effect size appears to be modest. Furthermore, the use of atypical antipsychotics in dementia is associated with an elevated risk of mortality and cerebrovascular events, along with metabolic effects, falls, EPSs, adverse cognitive effects, pneumonia and arrhythmias (114,115). These troublesome adverse effects should be considered prior to selecting quetiapine for the management of behavioral symptoms of dementia. Non-pharmacological measures should additionally be considered (116,117). Furthermore, a quetiapine discontinuation trial is warranted following a period of behavioral stabilization (114).

At least one meta-analysis supports the efficacy of adjunctive SGAs for the management of treatment-resistant depression (102). This review identified several RCTs (102) that support the efficacy of quetiapine augmentation for treatment-resistant depression; however, treatment with quetiapine may be associated with AEs, namely sedation, weight gain and metabolic imbalances (69). More recently, a number of RCTs have indicated that quetiapine may be effective for the treatment of MDD as a monotherapy (118). Safety and tolerability concerns should be considered.

In conclusion, this review challenges the traditional psychopharmacological concept of ‘class effects’. Quetiapine may have anti-anxiety and antidepressant effects beyond its antipsychotic efficacy. Evidence is more established for the use of quetiapine in GAD and MDD (both as an augmentation therapy for treatment-resistant depression and as a monotherapy). Furthermore, a beneficial effect for the management of agitation in dementia may exist, but the size of the effect appears to be modest. Studies on the other conditions reviewed in the present study possessed a number of methodological limitations that preclude any definite conclusions regarding the efficacy and safety of quetiapine. The present review suggests that quetiapine may have a pro-cognitive effect in schizophrenia, but the cognitive effects of quetiapine in BD require further investigation. This review has highlighted a number of important areas for further research. It is clear that large-scale RCTs on quetiapine are necessary to provide more robust evidence for the majority of the current off-label uses of quetiapine. Furthermore, studies should investigate the contribution of the neurotrophic and neuroprotective effects of quetiapine to some of its therapeutic effects, such as neurocognition.
